# Unambiguous detection of nitrated explosive vapours by fluorescence quenching of dendrimer films

**DOI:** 10.1038/ncomms9240

**Published:** 2015-09-15

**Authors:** Yan Geng, Mohammad A. Ali, Andrew J. Clulow, Shengqiang Fan, Paul L. Burn, Ian R. Gentle, Paul Meredith, Paul E. Shaw

**Affiliations:** 1 Centre for Organic Photonics & Electronics, The University of Queensland, Queensland 4072, Australia

## Abstract

Unambiguous and selective standoff (non-contact) infield detection of nitro-containing explosives and taggants is an important goal but difficult to achieve with standard analytical techniques. Oxidative fluorescence quenching is emerging as a high sensitivity method for detecting such materials but is prone to false positives—everyday items such as perfumes elicit similar responses. Here we report thin films of light-emitting dendrimers that detect vapours of explosives and taggants selectively—fluorescence quenching is not observed for a range of common interferents. Using a combination of neutron reflectometry, quartz crystal microbalance and photophysical measurements we show that the origin of the selectivity is primarily electronic and not the diffusion kinetics of the analyte or its distribution in the film. The results are a major advance in the development of sensing materials for the standoff detection of nitro-based explosive vapours, and deliver significant insights into the physical processes that govern the sensing efficacy.

## Abstract (web summary)

Selective standoff detection of explosives is challenging due to the presence of volatile interferents. Here, the authors report dendrimer thin films that display distinct fluorescence responses when exposed to explosives as opposed to common interferents, allowing selective detection of nitrated explosives.

## Graphical abstract

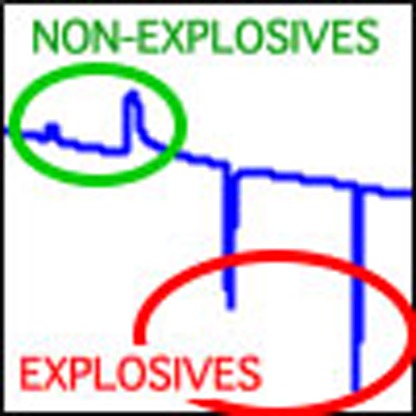

The capability to reliably detect explosive vapours is essential for the provision of security, the remediation of landmine fields in current and former warzones, as well as environmental monitoring. There are a number of detection instruments available based on standard analytical techniques (for example, ion mobility and mass spectrometry) but due to their inherently bulky nature and the need for swabbing to collect the sample, the use of such devices has largely been restricted to controlled screening environments and laboratories. As a consequence there is a large and unmet need for a compact portable device for infield use that enables rapid, standoff (non-contact sample collection), sensitive and selective detection of explosive vapours^1^,^2^. An emerging concept in this space has been the utilization of oxidative fluorescence quenching for the detection of nitro-group containing explosive materials. This has driven the development of fluorescent conjugated polymers^3^,^4^,^5^,^6^, supramolecular structures^7^,^8^,^9^, micelles^10^ and dendrimers^11^,^12^ capable of detecting vapours of nitroaromatic compounds such as 2,4,6-trinitrotoluene (TNT), which is the primary explosive in landmines and is part of numerous explosive compositions. However, an equally important but less studied area is the detection of plastic explosives. Due to the low volatility of plastic explosive compositions, taggants with higher vapour pressures, such as the nitroaliphatic compound 2,3-dimethyl-2,3-dinitrobutane (DMNB), are generally added to aid detection^13^,^14^.

At a superficial level, the design criteria for sensing materials for the detection of nitro-containing explosives are ‘well established'^4^,^15^. These criteria include the energetics of the system, which must be appropriate to ensure the target analyte molecule can accept an excited electron from the sensing material (see Fig. 1a for the general mechanism). In this regard, a way to gauge whether a material will be useful is to determine whether the difference between the reduction potentials of the analyte and the sensing material is greater than the exciton binding energy—if it is then sensing is in principle possible. Second, there needs to be an electrostatic affinity between the sensing material and the analyte, so that the binding enables electron transfer to readily occur. Third, films of the sensing material should be porous to enable the analyte vapours to be absorbed and diffuse. Fourth, a high solution affinity of a sensing material for an analyte will translate to a strong response to analyte vapour by a film of the same sensing material. Finally, it is thought that conjugated polymers are required for amplified spontaneous quenching, that is, where a single bound analyte can quench multiple chromophores leading to a gain in sensitivity. This gain in sensitivity is proposed to occur through exciton diffusion, whereby excitations migrate along the polymer chain until they encounter a bound analyte or decay^5^,^16^.

However, at a practical level these ‘established' criteria have a tendency to break down. For example, using energetics as the simple design criteria for a successful sensing material is not necessarily sufficient to achieve selectivity. It has been shown that the fluorescence of polymer and dendrimer sensing materials can be quenched by non-explosive materials even if the energetics for electron transfer appear to be unfavourable, leading to a critical lack of selectivity (Supplementary Fig. 1). In addition, it has been reported that fully dense dendrimer films can give rise to rapid detection^11^ showing that porosity is not an absolute requirement either, and that high solution affinity is not a reliable measure for determining the thin film sensing performance of a material^17^. Finally, dendrimer and not just polymer sensing materials can show ‘amplified quenching' where a single analyte can quench more than one chromophore^18^.

Thus, although oxidative fluorescence quenching has great promise for explosive sensing, the lack of selectivity has meant that detectors based on these materials cannot be widely deployed due to the problem of interferents creating false positive responses^5^,^8^,^19^. The ideal approach for achieving selectivity is that the responses to the desired analyte and potential interferent are orthogonal. That is, if the analyte quenches the fluorescence then the interferent must either not decrease the fluorescence or show an increase. If the response of the interferent and analyte are similar, for example, they both quench the luminescence, it will be extremely difficult to differentiate between them^5^,^20^, regardless of their quenching efficiency. That is, a low concentration of a strong quencher and a high concentration of a weak quencher can be expected to change the fluorescence in a similar manner. Indeed, small responses by interferents have sometimes been ignored or misinterpreted as a non-response^19^. Furthermore, if the interferent and analyte of interest both cause a decrease in fluorescence and are mixed it will not be possible to unambiguously determine whether a threat is present.

In this work we introduce light-emitting dendrimer films that are capable of real time selective sensing of nitrated analyte vapours. In the presence of nitro-containing analytes (TNT, 2,4-dinitrotoluene (DNT) and DMNB) the luminescence is quenched while in the presence of interferents the dendrimers exhibit either no change or an increase in their luminescence (Fig. 1b). We believe this is the first demonstration of selectivity towards nitroaromatics with a single-channel device. In the following results and discussion we use dendrimer **3** (Fig. 1c) as the exemplar material. We have investigated the origins of the selective response by considering the energetics of the system and probing the kinetics of the quenching process with broadband transient absorption spectroscopy. We also considered the interaction between the analyte vapours and the dendrimer thin film by using a combination of quartz crystal microbalance (QCM), neutron reflectivity and photoluminescence (PL) measurements to show that both nitro- and non-nitro-containing analytes can diffuse into the dendrimer sensing film, with only the nitro-containing analytes causing oxidative quenching of the fluorescence. Finally, in light of these findings we have reevaluated the design rules for sensing materials and now question the widely held belief that exciton diffusion always plays a first-order role in the detection process.

## Results

### Photophysics of the fluorescence-quenching process

Dendrimer **3** has a PL quantum yield (PLQY) of 67±7% in solution and 47±5% in the solid-state indicating that the first generation dendrons are sufficient to reduce the intermolecular interactions of the chromophores that would otherwise lead to the quenching of the luminescence in a film. For the study we used 4-nitrotoluene (pNT) as an analogue of a nitroaromatic explosive, DMNB as an example of an explosive taggant and naphthalene as an interferent. Naphthalene was chosen as the interferent as it is the active component in widely used mothballs, is a solid so is simple to handle yet has a high vapour pressure, is an aromatic molecule with no nitro-groups, and is readily available in the perdeuterated form required for the neutron reflectometry (NR) experiments.

To understand the photophysical properties of the quenching mechanism in detail we used broadband transient absorption spectroscopy (TAS), comparing the excited state kinetics of dendrimer **3** films with analyte-saturated films. Despite the extensive body of literature on the detection of nitroaromatics by fluorescence quenching there have been few systematic reports on the actual solid-state sensing mechanism^21^. Before undertaking TAS measurements on the films we cross-checked the solution singlet lifetime values of dendrimer **3** from TAS and time-correlated single photon counting, which were found to be the same within experimental error (∼1.15 and ∼1.23 ns, respectively). The decay of the singlet excited state measured with TAS in films of dendrimer **3** was fitted with a sum of two exponentials with an average lifetime of 390 ps (Supplementary Fig. 2), which is consistent with the lower film PLQY.

The photoexcited neat films of dendrimer **3** feature a bleach signal at wavelengths shorter than ∼480 nm and a broad feature at longer wavelengths that peaks at ∼590 nm (Fig. 2a), which is assigned to absorption by the singlet excited state. When the dendrimer film is saturated with pNT the initial excited state spectrum closely resembles that of a neat dendrimer **3** film (Fig. 2b). However, the singlet excited state is rapidly quenched within the first picosecond accompanied by the emergence of a second feature at shorter wavelengths with a peak at ∼505 nm (Fig. 3a). The same features are seen for DMNB although the quenching of the singlet excited state of dendrimer **3** is not as efficient (Supplementary Fig. 3). The film in a naphthalene-saturated atmosphere featured the same decay kinetics as observed in the neat film ( Fig. 3b).

### Analyte diffusion into sensing films

To gain insight into the ‘real time' diffusion process of each of the analytes into films of dendrimer **3**, we performed time-resolved QCM measurements with *in situ* PL monitoring. The data for pNT and naphthalene is shown in Fig. 4, with the data for DMNB in Supplementary Fig. 4. For pNT and naphthalene the introduction of the analyte vapour into a flowing stream of nitrogen led to a sudden change in the QCM frequency indicating rapid uptake of the analyte, with the response for DMNB being weaker. The uptake of pNT and DMNB led to quenching of the fluorescence while naphthalene gave a small increase (Fig. 4a,b and Supplementary Fig. 4). The mass uptake *M* of the analyte is related to the elapsed time *t* by


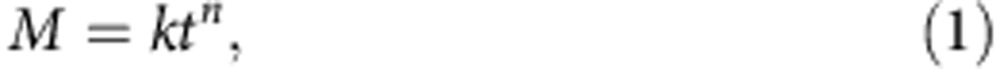


where *k* and *n* are constants^22^. By plotting ln(*M*) versus ln(*t*), *n* can be determined from the gradient (Supplementary Fig. 5). There are two clear sorption regimes: an initial fast stage followed by slower sorption. For both pNT and naphthalene *n*>1 during the fast stage (*n* is 1.8 for pNT and 1.9 for naphthalene), which corresponds to Super Case II diffusion^23^,^24^. As such, the dynamics of the analyte front are governed by the swelling kinetics of the film^25^. To first order the concentration of the analyte behind the front is constant so the mass uptake is proportional to the distance travelled by the front through the film. Equation 1 can thus be written as


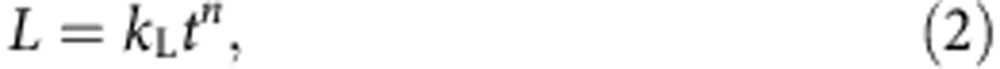


where *L* is the distance travelled by the front at time *t* and *k*_L_ is a constant. Differentiating equation 2 therefore yields an expression for the front velocity


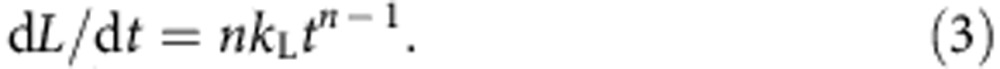


Thus for pNT, the front velocity is given by d*L*/d*t*=0.08*t*^0.8^ nm s^−1^ (*n*=1.8; *k*_L_ was found to be 4.5 × 10^−2^ nm s^−*n*^ by fitting with equation 2 to the the times corresponding to the point of inflection for films of different thickness (Supplementary Fig. 5)), that is, the diffusion rate increases with time.

We next consider how the analyte diffusion relates to the observed PL quenching shown in the inset of Fig. 4a. If the PL quenching efficiency, defined as


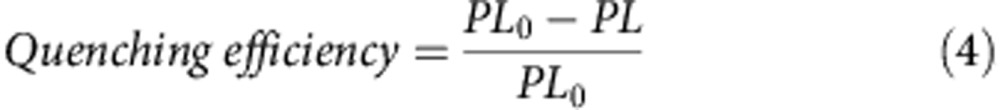


where *PL*_0_ is the photoluminescence intensity in the absence of analyte and *PL* is the intensity in the presence of analyte, is plotted with respect to the normalized mass uptake of the analyte it is clear that the responses of all the films are identical and independent of the film thickness (Fig. 5—note the mass used for normalization is taken from the saturated mass for each film thickness).

**Figure 1:**
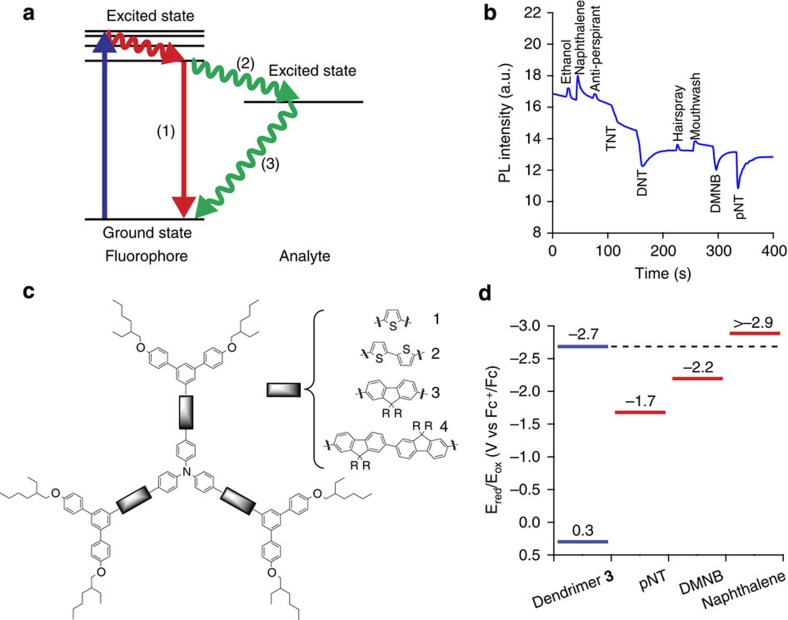
Principles of fluorescence quenching and selective sensing. (**a**) Schematic of the emissive decay of a fluorophore in the absence of an analyte (1) and in the presence of an analyte, where the excited state transfers an electron to the analyte (2), which then decays non-radiatively back to the fluorophore ground state (3). (**b**) The photoluminescence (PL) response of dendrimer **3** versus time with vapours of each analyte injected independently. It can be seen that the nitro-containing analytes (TNT, DNT=2,4-dinitrotoluene, DMNB and pNT=4-nitrotoluene) quench the PL whilst the vapours of a range of everyday interferents induce a rise of the PL, resulting in a selective response. Note TNT has a vapour pressure of 5 ppb and the strong response to this concentration illustrates the sensitivity of the sensing films. (**c**) Examples of dendrimers that show selective sensing for nitrated analytes. R=*n*-propyl. (**d**) The reduction potentials of dendrimer **3** and the analytes.

**Figure 2:**
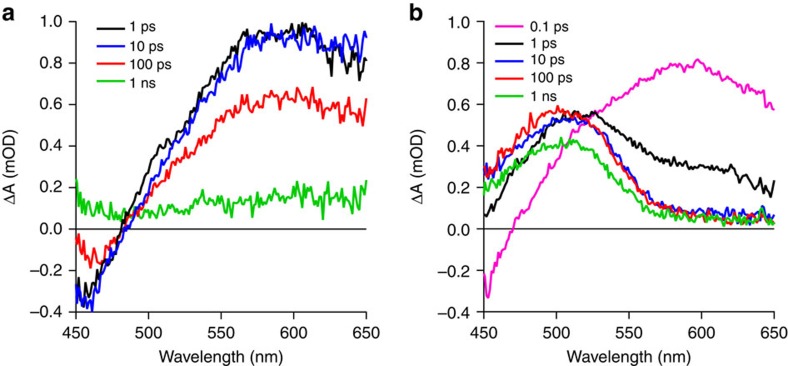
Transient absorption spectra. Transient absorption spectra for the (**a**) as-cast dendrimer film and (**b**) the dendrimer film saturated with pNT vapour.

Finally, to further probe the diffusion of the analytes into the dendrimer thin films we performed NR, which allows the vertical structure of thin films relative to the substrate, that is, thickness and mass density, to be determined. One of the key advantages of NR over complementary techniques, such as X-ray reflectometry and ellipsometry, is that contrast between organic compounds with similar mass density can be achieved with selective deuteration^26^. In this case, perdeuterated analytes were used to contrast with the protonated dendrimer **3** films. Measuring and fitting to the neutron reflectivity profiles of an as-cast dendrimer **3** film and when saturated with deuterated analyte vapour allowed the concentration and distribution of the analyte to be determined. It is important to note that the QCM measurements and NR were undertaken under different conditions. The NR was performed under steady-state conditions on analyte-saturated films, whilst the QCM measurements were performed under dynamic exposure to diluted analyte vapours with the films reaching equilibrium during the experiment. Although it is possible to do time-resolved NR^27^, the rapidity of the PL change observed in Fig. 1b is faster than the time resolution of the NR experiment. Fig. 6 shows the modelled scattering length density (SLD) versus film thickness profiles for the films of dendrimer **3** before and after reaching equilibrium in a saturated analyte atmosphere with the corresponding PL of the film in the inset (the corresponding neutron reflectivity profiles are in Supplementary Fig. 6). From the measurements, the SLD of dendrimer **3** was determined to be 1.11±0.02 × 10^−6^ Å^−2^, corresponding to a mass density of 1.01±0.02 g cm^−3^, which is typical of a fully dense organic film. It can be seen that the SLD of the d-pNT saturated film (Fig. 6a and Supplementary Fig. 6a) has increased and is uniform across the film after it has swollen to accommodate the d-pNT vapour. The ratio of d-pNT:dendrimer **3** in the saturated film was determined to be 1:2.2, which as can be seen from the inset, leads to complete quenching of the PL. A similar outcome was observed for d-DMNB (Fig. 6b and Supplementary Fig. 6b)—the analyte was again evenly distributed throughout the swollen film with a d-DMNB:dendrimer **3** ratio of 1:3.4. Whilst the changes in the case of d-naphthalene are smaller, the NR suggests that analyte again diffuses throughout the film with an even distribution (Fig. 6c and Supplementary Fig. 6c). The PL of the film was found to increase slightly (Fig. 6c), as was observed in the real time measurement shown in Fig. 1b and the PL data for the QCM measurements in Fig. 4b. Thus, the film displays an affinity for naphthalene vapours, which are able to diffuse throughout (the d-naphthalene:dendrimer **3** ratio at saturation was 1:9.0, which was lower than that of the nitro-containing analytes) although the thickness of the film did not change significantly.

## Discussion

The family of light-emitting dendrimers that shows selectivity (Fig. 1b) for nitro-containing analytes are comprised of a triphenylamine moiety at the centre, first generation biphenyl dendrons and 2-ethylhexyloxy surface groups (Fig. 1c). Selectivity was contingent on the presence of the central triphenylamine moiety and independent of the (hetero)aryl units linking to the dendrons. The compounds were created based on the energetics and electrostatic design rules to give high sensitivity^28^, and their syntheses are described in the Supplementary Information.

The absence of quenching by the naphthalene vapours shown in Fig. 1b could be due to unfavourable energetics, thermodynamics or simply that the vapours do not enter the film. We will first consider the energetics of the system and the photophysical properties of the oxidative quenching process. The reduction potential of dendrimer **3** was estimated from its solution oxidation potential in combination with the PL and absorption spectra following the method by Yu *et al.*^29^ for thin films. The reduction potentials of dendrimer **3**, pNT, DMNB and naphthalene were −2.7 V, −1.7 V (ref. 30), −2.2 V (ref. 30) and ≥−2.9 V, respectively (Fig. 1d). These results indicate that electron transfer would be expected between dendrimer **3** and both pNT and DMNB but not naphthalene, which is consistent with the absence of fluorescence quenching by naphthalene vapour whether or not it had diffused into the film.

**Figure 3:**
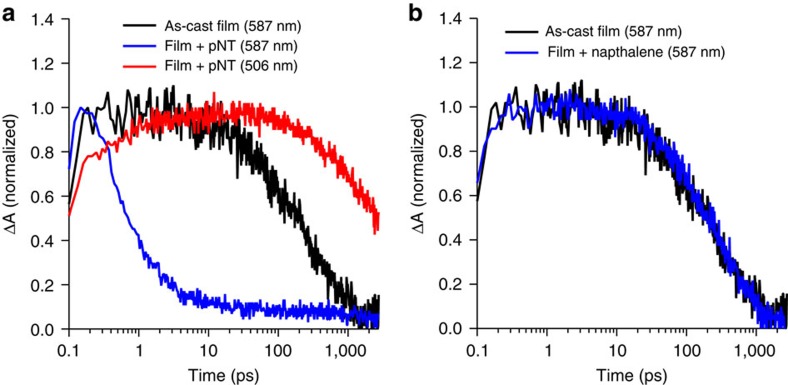
Transient absorption spectroscopy kinetics. (**a**) Excited state kinetics of the singlet state (587 nm) and charge transfer state (506 nm) in the as-cast dendrimer film and when saturated with pNT. (**b**) Kinetics of the singlet state in the as-cast dendrimer film and when saturated with naphthalene.

**Figure 4:**
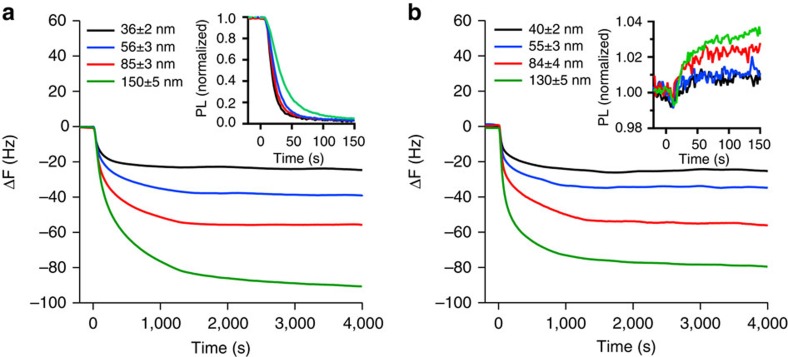
Analyte vapour sorption. Change in QCM frequency with time following the injection of (**a**) pNT and (**b**) naphthalene. The normalized change in the PL intensity is shown in the corresponding inset. The frequency response curves are the average from measurements on three films.

**Figure 5:**
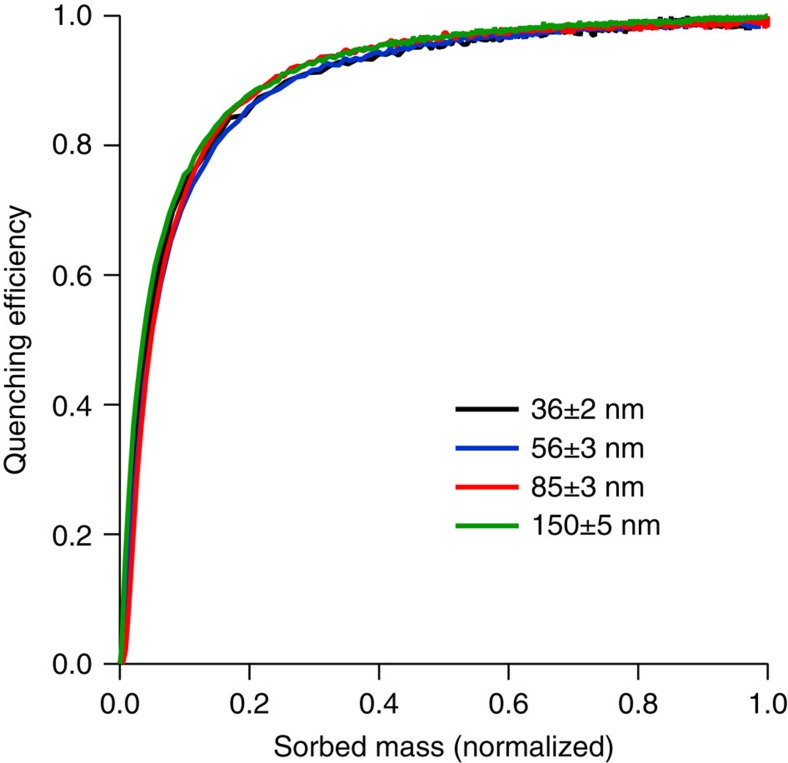
PL quenching efficiency of dendrimer 3 films. The quenching efficiency relative to the normalized mass uptake of pNT for dendrimer films with a range of different thicknesses. The quenching efficiency curves are the average from measurements on three films.

**Figure 6:**
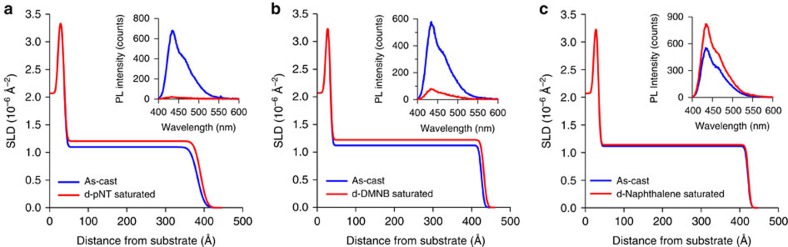
Analyte vapour distribution. Modelled SLD versus thickness plots for films of dendrimer **3** before and after saturation with (**a**) d-pNT, (**b**) d-DMNB and (**c**) d-naphthalene. Insets are the corresponding PL spectra of the films.

Turning now to the TAS experiments, the extremely fast quenching of the singlet excited state observed is comparable to that measured in fullerene-containing blends used in organic solar cells where electron transfer occurs on a femtosecond timescale. The fact that the singlet excited state appears to be fully quenched within the first 10 ps suggests a high concentration of pNT within the film and/or that exciton diffusion is very efficient. Fitting confirms that the decay of the singlet excited state matches the rise of the state absorbing at ∼505 nm (Fig. 3a) and, hence, this feature is a product of the quenching process. The decay of the charge-transfer state is exponential with a lifetime of ∼7 ns for pNT, suggesting that relaxation of this state is via geminate recombination. Since the peak at 505 nm is the same for both nitrated analytes, pNT and DMNB (although the latter is weaker due to the lower driving force for the electron transfer process—see Fig. 1d), it is logical to conclude that it arises from the same state. As pNT and DMNB have different electron affinities (Fig. 1d) it is unlikely they would feature excited state transitions of the same energy when reduced. The state is therefore assigned to an absorption of the oxidized dendrimer, which given that it is higher in energy than the singlet absorption, is due to an excitation to a higher singlet state rather than polaron absorption. The fact that the naphthalene-saturated film has the same TAS signature as the as-cast film is consistent with charge transfer not being energetically favourable, but also indicates that the presence of the naphthalene does not significantly change the radiative and non-radiative decay rates of dendrimer **3**. Hence, the increase in the fluorescence signal observed when dendrimer **3** was exposed to naphthalene vapour is not due to a change in the photophysical properties of the sensing molecules but a bulk effect on the optical properties of the film. The TAS measurements are thus very informative about the energy criteria for sensitive and selective sensing but they do not provide information concerning the diffusion and concentration of the analyte vapours in the dendrimer film, and the role this plays in the sensing process.

The QCM measurements showed that on introduction of the nitrated analytes there was a rapid uptake of mass by the sensing films. Increasing the film thickness resulted in an increase in the mass uptake, which indicates that the analytes were being absorbed by the film rather than adsorbed onto the surface. For both pNT and naphthalene Super Case II diffusion was observed. Super Case II diffusion is where a film swells as another material diffuses into it (in our case the analyte), with the diffusing material propagating as an accelerating front through the film. As such the dynamics of the analyte front are governed by the swelling kinetics of the film^25^. By calculating the front velocity and assuming that the point of inflection indicates completion of the Super Case II transport mode (Supplementary Fig. 5) we find that the diffusion rate increases with time so even for a 150 nm film the diffusional front would reach the substrate within 2 min. Thus these measurements provide valuable insights into the interaction of the analytes with the bulk of the film and in particular the diffusion process. As such, they are also instructive for optimising the film thickness to gain the maximum fluorescence signal relative to response time. A final aspect of the combined QCM–PL measurement was that the thickness independence of the quenching efficiency is consistent with Super Case II diffusion and a sharp diffusion front propagating through the film behind which the penetrant concentration is uniform. The thickness independence could in principle also be explained by having a uniform distribution of analyte throughout the film, which increases in concentration with time, but this scenario is unlikely to occur in thicker films. Since the mass uptake is proportional to the distance travelled through the film and assuming a sharp diffusion front, the normalized mass uptake corresponds to the extent of propagation of the front through the film. Taking the point of inflection (Supplementary Fig. 5) as corresponding to the arrival of the front at the substrate, the concentration of pNT behind the front was calculated to be (4.9±0.8) × 10^−2^ nm^−3^, which implies that the maximum centre-to-centre distance between dendrimer **3** and pNT is just 2.7±0.2 nm (1.0 nm radius for dendrimer **3**). The short centre-to-centre dendrimer-analyte distance observed by QCM is consistent with the TAS measurements, which show that electron transfer occurs on a timescale that is much shorter than that required for typical exciton diffusion lengths. It has been previously thought that relatively long exciton diffusion lengths were required to achieve effective quenching^16^. However, if the analyte is moving through the film as a well-defined front, as in the case here, then long diffusion lengths are simply not needed and hence the design of sensing materials around this parameter may be unnecessary.

Finally, the steady-state NR measurements showed that all three analytes were evenly distributed throughout the sensing film, which is consistent with the Super Case II diffusion observed from the QCM measurements. This is in contrast to a concentration gradient as was previously observed for pNT saturated bifluorene-cored dendrimer sensing films^11^.

In conclusion, we have developed a series of dendrimers for sensing in the solid-state that are selective for nitro-containing analyte vapours, which is a significant advance in the detection of explosive vapours with fluorescence. The fluorescence from the films was quenched when exposed to vapours of nitro-containing compounds but crucially when films were exposed to a range of interferent vapours, the fluorescence was unchanged or increased, thus providing an unambiguous response to potential explosive threats. Both QCM and neutron reflectivity measurements showed that nitroaromatic (pNT), nitroaliphatic (DMNB) and aromatic (naphthalene) vapours could diffuse into the dendrimer film showing that dendrimer-analyte affinity was not the origin of the lack of fluorescence quenching by naphthalene. The QCM measurements combined with *in situ* PL measurements showed that all the analytes tested diffused into the dendrimer film via a Super Case II mechanism, whereby a high analyte concentration front propagates through the film. Critically the analyte concentration behind the front was sufficiently high such that the average dendrimer-analyte separation was much less than typical exciton diffusion lengths. Coupled with the fact that a charge-transfer state formed on the femtosecond timescale, this indicates that exciton diffusion does not play a significant role in the efficient fluorescence quenching observed. This is a key insight as it provides a new direction for designing sensing materials for the detection of vapours in the solid state, and highlights the importance of understanding the nature of the interaction at the solid–vapour interface. Finally, dendrimers containing triphenylamine centres are the first class of materials in which the response to nitro-containing analytes is dominated by the energy design criteria. The electron affinity of the naphthalene was insufficient to oxidize the photoexcited dendrimers whereas the nitro-containing analytes were able to oxidatively quench the fluorescence. Importantly, detection of the nitro-containing analytes was found not be masked by the interferent used in this work, even when the vapour pressure of the interferent was 100 times greater than that of the nitro-containing analyte (Supplementary Fig. 7).

## Methods

### PL quantum yield

The solution PLQYs of the dendrimers were measured relative to a reference of quinine sulfate solution in 0.5 M sulfuric acid^31^. The excitation wavelength was 360 nm with the measured emission spectra corrected for self-absorption. All solutions were prepared in spectroscopic grade tetrahydrofuran with no degassing. The film PLQY measurements were performed using the method described by Greenham *et al.*^32^. The 325 nm output of a HeCd laser was attenuated with neutral density filters to ∼0.2−0.3 mW and used to photoexcite the films. The interior of the integrating sphere was flushed with nitrogen to minimize photodegradation, and the PL signal was measured with a calibrated photodiode. The PLQY was measured at multiple points on each film and averaged.

### Transient absorption spectroscopy

The dendrimer was spin-coated onto fused silica substrates, which were loaded into a sealed optical chamber in a nitrogen-filled glove box to minimize photo-oxidation of the samples during the measurement. The measurements on the vapour-exposed films were performed with a second optical chamber with a small cap containing the analyte placed inside the chamber. Once the sample was loaded into the optical chamber the interior was flushed with nitrogen and then left to rest so that the atmosphere inside the chamber was saturated for the measurements. The broadband transient absorption measurements were performed with a HELIOS transient absorption spectrometer (Ultrafast Systems, LLC). The output from a Spectra-Physics Spitfire amplifier operating at 1 kHz was split to seed a TOPAS optical parametric amplifier and generate the 380 nm pump beam, with the remainder used to generate the white light continuum for the probe. The intensity of the pump beam was attenuated with a neutral density filter until the decay kinetics of the as-cast film showed no power dependence. The transient absorption data were corrected for chirp before analysis.

### QCM measurements

The QCM measurements were undertaken with a custom-built experimental setup^33^ at an ambient temperature of 22.5±1.0 °C. The chamber comprises a QCM holder and gas-mixing compartment with the carrier gas (nitrogen) supplied by two mass flow controllers. One line enters directly into the gas-mixing compartment (nitrogen only) with the second line passes through a coil coated with the chosen analyte to introduce the analyte vapour into the nitrogen stream. Mixing of the gas streams is aided by baffles in the mixing chamber, which also minimize disturbance of the QCM by the gas stream. The QCM chip was sandwiched between two O-rings within a Teflon holder, which secured its position and reduced vibration and drift. The diameters of the quartz crystal and gold electrode were 13.7 and 5.11 mm, respectively. The CH Instruments Model 400B utilizes a time-resolved mode to measure the frequency difference between the working crystal (7.990–7.955 MHz) and the reference crystal oscillations (8.000 MHz). The Sauerbrey equation was used to estimate the mass changes (Δ*M*) from the frequency changes (Δ*f*) of the crystal output given by


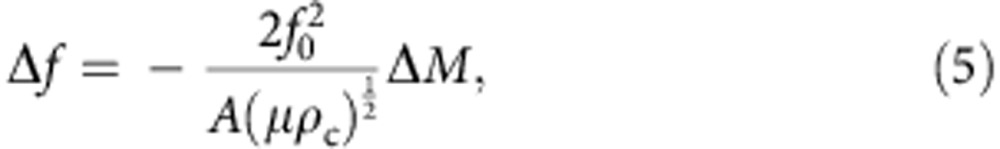


where *f*_0_ is the resonant frequency of the fundamental mode of the crystal, *A* is the area of the gold disk coated onto the crystal (0.205 cm^2^), *ρ*_c_ is the density of the crystal and *μ* is the shear modulus of quartz. The density of the crystal was 2.648 g cm^−3^ and the shear modulus was 2.947 × 10^11^ g cm^−1^ s^−2^ (data provided by CH Instruments, Inc.). Therefore, for an 8 MHz crystal, a 0.1 Hz change in frequency corresponds to a mass change of 0.14 ng. However, it is important to note that QCMs are very sensitive to the environment including local vibrations and temperature changes. We therefore measured the QCM drift under nitrogen at a flow rate of 1,500 ml min^−1^ and found that the frequency varied by up to ±5 Hz during 6,000 s running time, which corresponds to an uncertainty of ±7 ng in the measurements. It is important to note that this uncertainty in the mass is greater than the differences observed in separate measurements under the same conditions, and hence sets the upper limits on the errors. Each sorption experiment was repeated three times and average values taken for further calculation. An Xdip-SV1 dip-coater was employed for coating the QCM chip. The QCM chip was first cleaned using toluene, followed by rinsing with ethanol and acetone before being dried with a stream of nitrogen. It was then immersed in a 10 or 15 mg ml^−1^ solution of the dendrimer in toluene and withdrawn from the bath at a prescribed withdrawal speed to control the final film thickness. The process was done under ambient conditions. The film was then left to dry in air. Thicknesses of the films coated on the QCM crystal were determined from the optical density and absorption coefficient, and crosschecked with the thickness expected from the mass and film density^33^. To determine the absorption coefficient, the films were spin-coated on fused silica substrates and thicknesses were measured using a Veeco Dektak 150 surface profilometer and the absorptions were measured using a Varian Cary 5000 spectrophotometer. The concentrations of analytes used in the experiment were 15 p.p.m., 11 p.p.m. and 1 p.p.m. at a flow rate of 1,500 ml min^−1^ for the pNT, naphthalene and DMNB, respectively.

### Neutron reflectometry

Neutron reflectometry was performed using the Platypus time-of-flight neutron reflectometer and a cold neutron spectrum (2.8 Å<*λ*<18.0 Å) at the OPAL 20 MW research reactor (Australian Nuclear Science and Technology Organisation (ANSTO), Sydney, Australia)^34^. Neutron pulses (24 Hz) were generated using a disc chopper system (EADS Astrium GmbH) in the medium resolution mode (Δ*λ*/*λ*=4%) and recorded on a two-dimensional helium-3 neutron detector (Denex GmbH). Reflected beam spectra were collected at 0.7° for 1,800 s and 2.5° for 7,200–8,000 s and direct beam measurements were collected under the same collimation conditions for both reflection angles. Experiments were conducted in a custom-built sample chamber in air, which incorporated a 365 nm LED (Nichia) light source and an optical fibre coupled to an Ocean Optics USB2000 spectrograph for the *in situ* PL measurements. The reflectivity profiles of the as-cast films were recorded before they were sealed in jars charged with perdeuterated analyte under cotton wool to prevent solid analyte coming into contact with the films. The films were allowed to saturate with analyte for a minimum of 5 h before their reflectivity profiles were again recorded. To maintain saturation of the films with analyte throughout the neutron measurement the sample chamber was charged with cotton wool coated with the appropriate perdeuterated analyte. Analysis of the reflectivity profiles was performed using the Motofit reflectometry analysis program^35^. A description of the method for determining the analyte:dendrimer ratio in the saturated films is given in the Supplementary Information. All of the fits described used an SLD of 2.07 × 10^−6^ Å^−2^ for the silicon substrate and included a native oxide layer on the surface of the substrate with an SLD of 3.47 × 10^−6^ Å^−2^. The errors in the modelled thickness, roughness and SLDs of the films given in Supplementary Table 1 are the s.d. determined by the Motofit analysis software. When calculating the average SLD of the as-cast dendrimer films and the corresponding dendrimer density these errors were propagated using the chain rule.

Silicon wafers of 50 mm diameter were cleaned in Piranha solution (a mixture of sulfuric acid (98%, 245 ml) and hydrogen peroxide (30%, aq, 105 ml)) before deposition of the dendrimer films by spin-coating from toluene solutions with a concentration of 10 mg ml^−1^.

## Additional information

**How to cite this article:** Geng, Y. *et al.* Unambiguous detection of nitrated explosive vapours by fluorescence quenching of dendrimer films. *Nat. Commun.* 6:8240 doi: 10.1038/ncomms9240 (2015).

## Supplementary Material

Supplementary Information Supplementary Figures 1-7, Supplementary Table 1, Supplementary Methods and Supplementary References
