# Supplementary material for: Unambiguous detection of nitrated explosive vapours by fluorescence quenching of dendrimer films
Source: Nat Commun. 2015 Sep 15;6:8240. doi: 10.1038/ncomms9240 (PMC4579782; doi:10.1038/ncomms9240)
Supplement: Supplementary Information — Supplementary Figures 1-7, Supplementary Table 1, Supplementary Methods and Supplementary References [file ncomms9240-s1.pdf]

Supplementary Information

Supplementary Figures

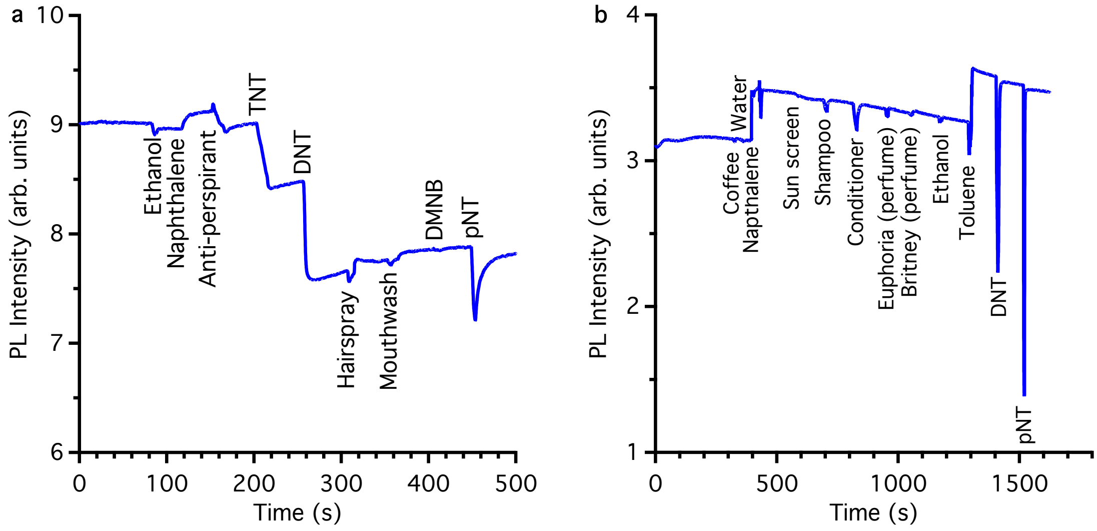

Supplementary Figure 1 | Examples of sensing responses with poor selectivity. PL versus time with each analyte injected independently and the PL response monitored. It can be seen that the nitro-containing analytes (TNT, DNT, DMNB and pNT) quench the PL as do a range of common non-nitro-containing vapours and interferents for (a) an iptycene-based polymer[1] and (b) first generation bifluorene-core dendrimer[2].

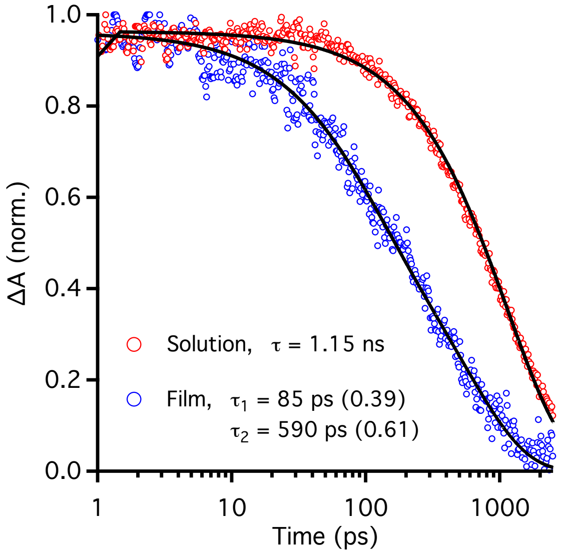

Supplementary Figure 2 | Excited state decay of dendrimer 3. Singlet state decay of the dendrimer in solution and film measured with TAS. Fits are shown as solid black lines with the fit parameters included in the legend.

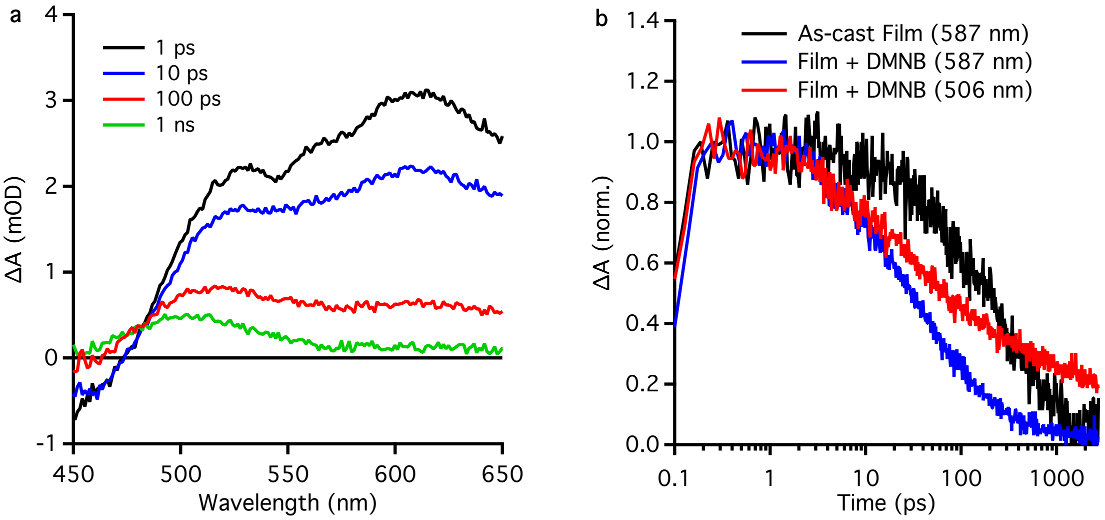

Supplementary Figure 3 | Transient absorption spectroscopy of dendrimer 3 films saturated with DMNB. (a) Excited state absorption for a film of dendrimer **3** saturated with DMNB vapour, and (b) the decay kinetics of the singlet exciton (587 nm) and charge-transfer (506 nm) state.

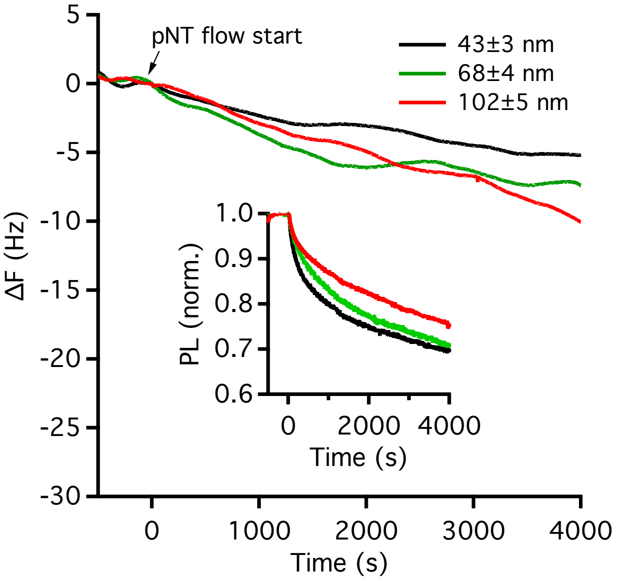

Supplementary Figure 4 | DMNB vapour absorption by dendrimer 3 films. The change in QCM frequency with time following exposure to DMNB vapour is shown with the normalised change in the PL intensity in the inset. The frequency response curves are the average from measurements on three films.

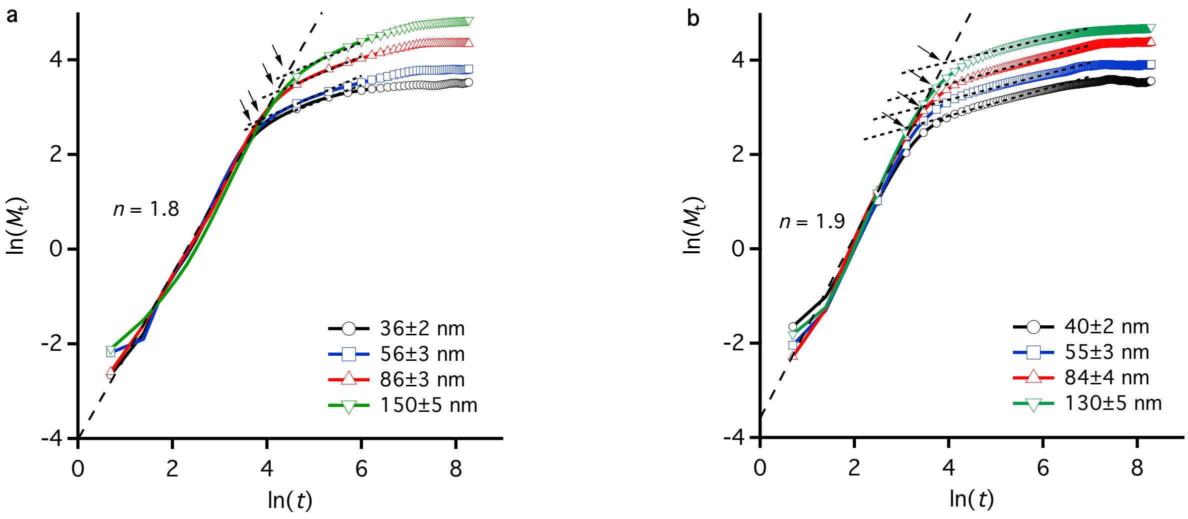

Supplementary Figure 5 | Identification of the analyte absorption process by dendrimer 3 films. Plot of ln(*M*) vs ln(*t*) for (a) pNT and (b) naphthalene. Arrows indicate points of inflection.

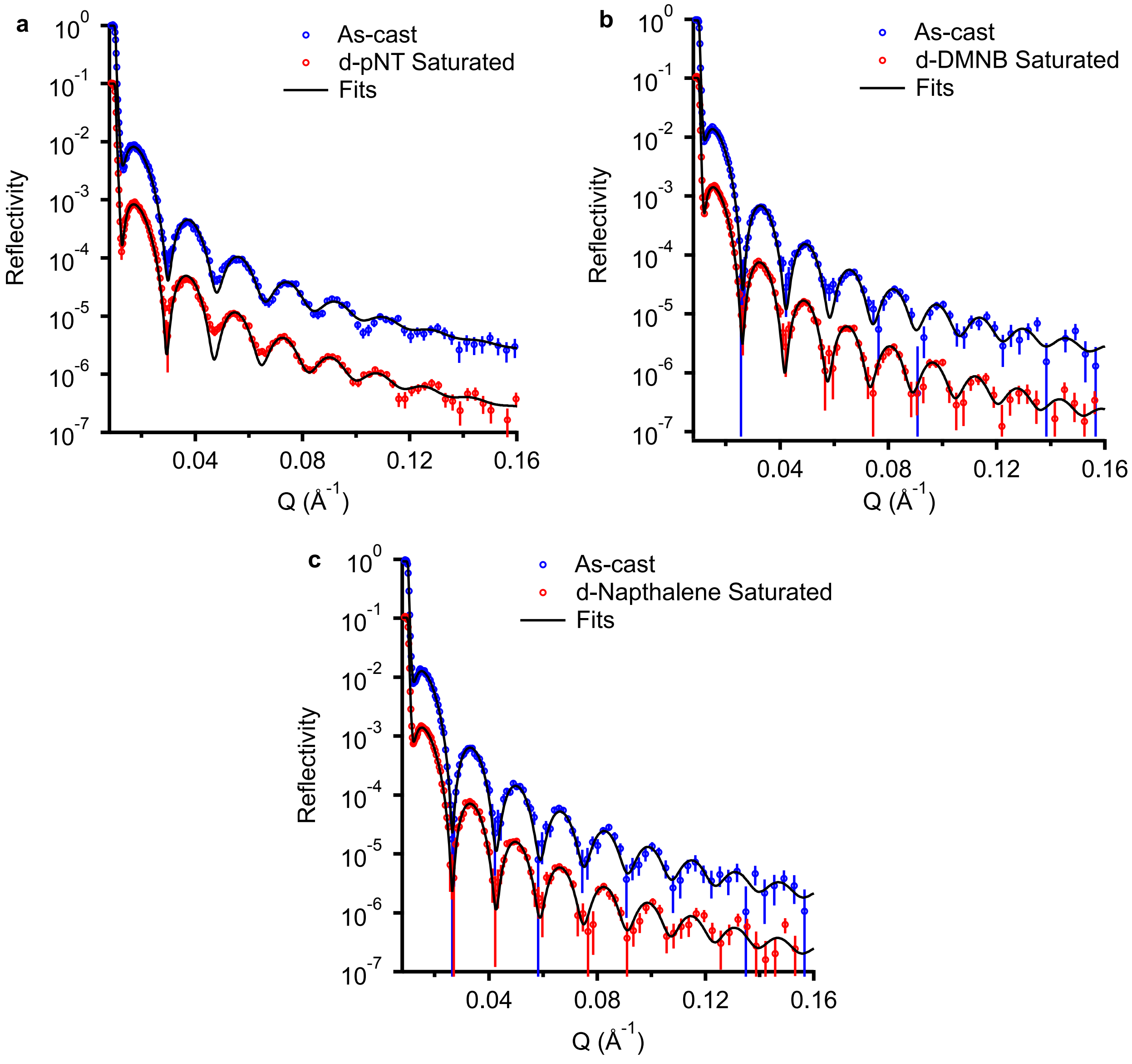

Supplementary Figure 6 | Neutron reflectivity profiles and associated fits. Neutron reflectivity

profiles and model fits (parameters listed in Supplementary Table 1) corresponding to the data in Figure 6 for films of dendrimer **3** with (a) d-pNT, (b) d-DMNB and (c) d-naphthalene. Traces are offset for clarity.

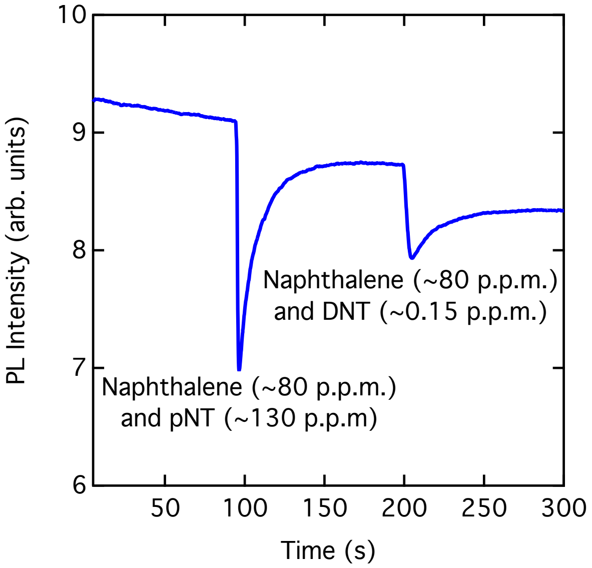

Supplementary Figure 7 | Example response of a dendrimer 3 film to nitro-containing:interferent vapour mixtures. The photoluminescence (PL) response of dendrimer **3** to vapours of pNT (~130 ppm) and DNT (~0.15 ppm) mixed with naphthalene (~80 ppm).

Supplementary Tables

Supplementary Table 1 | Summary of parameters for the neutron reflectivity profile fits. Model fitting parameters and associated errors determined using the Motofit analysis program and analyte:dendrimer ratios calculated as described in the Supplementary Methods.

Analyte	Film Condition	Thickness (Å)	SLD ($\times 10^{-6}$ Å^{-2})	Roughness (Å)	Analyte:Dendrimer Ratio
d-pNT	As-cast	346 ± 1	1.10 ± 0.01	14 ± 1	N/A
	Saturated	353 ± 1	1.20 ± 0.01	13 ± 1	1 : 2.2
d-DMNB	As-cast	392 ± 1	1.12 ± 0.01	6 ± 2	N/A
	Saturated	398 ± 1	1.22 ± 0.01	6 ± 2	1 : 3.4
d-naphthalene	As-cast	387 ± 1	1.11 ± 0.01	5 ± 2	N/A
	Saturated	388 ± 1	1.14 ± 0.01	5 ± 2	1 : 9.0

Supplementary Methods

Synthesis of 1 and 2

The syntheses of **1** and **2** have been reported elsewhere[3]. Solvents were distilled before use. When solvent mixtures are reported, the proportions are given by volume. Dichloromethane (CH_2Cl_2) used for the electrochemical experiments was dried over calcium hydride, distilled, and then stored over activated 4 Å molecular sieves. Tetrahydrofuran (THF) used for anhydrous reactions was stirred over sodium/benzophenone and then distilled immediately prior to use. THF used for electrochemical experiments was then further distilled from lithium aluminium hydride immediately prior to use. ^{1}H NMR spectra were recorded on a Bruker Avance 500 (500 MHz) or 400 (400 MHz) spectrometer. Chemical shifts are reported in parts per million (ppm) and are referenced to the residual solvent peak (chloroform, ^{1}H = 7.26 ppm, ^{13}C = 77.0 ppm). Coupling constants (J) are given in hertz (Hz) and are quoted to the nearest 0.5 Hz. Peak multiplicities are described in the following way: doublet (d), multiplet (m); peak identities are described as Et = 2-ethylhexyloxy H, bpH = branching phenyl H, spH= surface phenyl H, FH = fluorenyl H, and TPAH = triphenylamine H. Mass spectra were recorded on an Applied Biosystems Voyager-DE STR instrument with a matrix-assisted laser-desorption ionisation-time of flight (MALDI-TOF MS; positive-ion, reflectron) setup, with *(E)*-2-[3-(4-*t*-butylphenyl)-2-methyl-2-propenylidene] malononitrile (DCTB) as the matrix. Loading of the sample on the analysis stage was done by first spotting a solution of matrix in dichloromethane onto the plate, and when dried, a solution of dendrimer in dichloromethane-light petroleum ether mixture (1:1) was spotted, and finally the matrix solution was deposited on top. Infrared spectra were recorded on a Perkin-Elmer Spectrum 100 FT-IR spectrometer. Thermal gravimetric analyses were performed with a Perkin-Elmer STA 6000 under nitrogen, and results are reported as the temperature at which a 5% weight loss ($T_{5\%}$) was observed. Differential scanning calorimetry was performed with a Perkin-Elmer Diamond DSC. Elemental analyses were carried out in the Microanalysis Facility of the School of Chemistry and Molecular Biosciences, The University of Queensland.

Synthesis of 3

*7-[3,5-Bis(4-{2-ethylhexyloxy}phenyl)phenyl]-2-bromo-9,9-bis-*n*-propylfluorene.*

A mixture of 2-bromo-7-iodo-9,9-bis-*n*-propylfluorene[4] (613 mg, 1.35 mmol), 3,5-bis[4-(2-ethylhexyloxy)phenyl]phenyl-4,4,5,5-tetramethyl-1,3,2-dioxaborolane[5] (455 mg, 0.74 mmol), tetrakis(triphenylphosphine)palladium(0) (58 mg, 0.05 mmol), toluene (15 mL), *t*-butanol (4 mL) and aqueous sodium carbonate (2 M, 10 mL) was placed under vacuum and then backfilled with nitrogen three times before being heated in an oil bath at 60 ºC for 24 h. After cooling to room temperature, the layers were separated. The aqueous phase was extracted with ethyl acetate (3 × 20 mL), and the combined organic phases were washed with brine (2 × 20 mL), dried over magnesium

sulfate, filtered, and then the solvent was removed. The residue was purified by column chromatography over silica using dichloromethane:hexane (1:5) as eluent to afford *7-[3,5-Bis(4-{2-ethylhexyloxy}phenyl)phenyl]-2-bromo-9,9-bis-*n*-propylfluorene* as a light yellow oil (620 mg, 76%). Elemental analysis (%) calcd for $C_{53}H_{65}BrO_2$ C 78.2, H 8.05; Found: C 78.25, H 8.2. ^{1}H NMR (δ 400 MHz, $CDCl_3$): 7.75 (1 H, d, J = 8, FH) 7.73 (2 H, d, J = 1.5, bpH), 7.70 (1 H, dd, J =1.5, J = 1.5, bpH), 7.63-7.67 (5 H, m, FH and spH), 7.61 (1 H, d, J = 1, FH), 7.59 (1 H, d, J = 8, FH), 7.49 (1 H, d, J = 1.5, FH), 7.47 (1 H, dd, J = 1.5, J = 8, FH), 7.03 (4 H, 1/2AA'BB', spH), 3.91-3.92 (4 H, m, OCH_2), 1.91-2.06 (4 H, bm, $PrCH_2$), 1.72-1.81 (2 H, m, EtCH),1.31-1.60 (16 H, m, Et CH_2), 0.91-0.98 (12 H, m, $EtCH_3$), 0.70 (10 H, bm, $PrCH_2$ and $PrCH_3$). ^{13}C NMR(δ 100 MHz, $CDCl_3$): 159.3, 153.3, 151.1, 142.5, 142.2, 140.8, 139.8, 139.5, 133.6, 130.1, 128.4, 126.4, 126.3, 124.6, 124.3, 121.8, 121.1, 120.1, 114.9, 70.6, 55.8, 42.7, 39.5, 30.6, 29.1, 23.9, 23.1, 17.2, 14.5, 14.2, 11.2. λ_{max} (CH_2Cl_2)/nm: 284 (logε /dm^3 mol^{-1} cm^{-1}: 4.90), 321 (4.74). MS (MALDI-TOF, DCTB) Anal.Calcd for $C_{53}H_{65}BrO_2$: 812.4 (100%), 813.4 (58%), 814.4 (97%), 815.4 (57%), 816.4 (17%). Found: 812.4 (100%), 813.4 (66%), 814.4 (99%), 815.4 (61%), 816.4 (20%).

*7-[3,5-Bis(4-{2-ethylhexyloxy}phenyl)phenyl]-2-(4,4,5,5,-tetramethyl-1,3,2-dioxaborolanyl)-9,9-bis-*n*-propylfluorene*

A solution of 7-[3,5-bis(4-{2-ethylhexyloxy}phenyl)phenyl]-2-bromo-9,9-bis-*n*-propylfluorene (1.22 g, 1.50 mmol) in tetrahydrofuran (25 mL) under nitrogen was cooled in a dry ice/acetone bath. *n*-Butyllithium (1.6 M in hexane, 1.12 mL, 1.79 mmol) was added and the reaction was stirred for 10 min. 2-*iso*-Propoxy-4,4,5,5-tetramethyl-1,3,2-dioxaborolane (0.46 mL, 2.25 mmol) was added, and the reaction was stirred for 20 min before being allowed to warm to room temperature, after which is was stirred for a further 16 h. The mixture was finally stirred at 55 °C for 2 h before being allowed to cool to room temperature. Water (10 ml) was added and the layers were separated. The aqueous phase was extracted with ethylacetate (3 × 20 mL), and the combined organic phases were washed with brine (2 × 20 mL), dried over magnesium sulfate, filtered, and then the solvent was removed. The residue was purified by dissolution into dichloromethane (5 mL) followed by pouring into methanol (60 mL). The precipitate was recovered by centrifugation (3000 rpm, 5 min), and dried to give *7-[3,5-bis(4-{2-ethylhexyloxy}phenyl)phenyl]-2-(4,4,5,5,-tetramethyl-1,3,2-dioxaborolanyl)-9,9-bis-*n*-propylfluorene* as a white solid (1.05 g, 81%). Elemental analysis (%) calcd for $C_{59}H_{77}BO_4$ C 82.3, H 9.0; Found: C 82.3, H 9.2. ^{1}H NMR (δ400 MHz, $CDCl_3$): 7.77-7.84 (3 H, m, FH) 7.72-7.74 (3 H, FH and bpH), 7.70 (1 H, dd, J = 1.5, J = 1.5, bpH) 7.62-7.68 (6 H, m, FH and spH), 7.03 (4 H, 1/2AA'BB', spH), 3.91-3.92 (4 H, m, OCH_2), 1.97-2.10 (4 H, m, PrCH2), 1.72-1.82 (2 H, m, EtCH), 1.31-1.57 (28 H, m, BCH_3 and $EtCH_2$), 0.90-0.98 (12 H, m, $EtCH_3$), 0.67 (10 H, m, $PrCH_2$ and $PrCH_3$). ^{13}C NMR(δ100 MHz, $CDCl_3$): 159.2, 152.1, 150.2, 143.7, 142.7, 142.1, 140.8, 140.3, 133.8, 133.6, 128.9, 128.4, 126.2, 124.4, 124.3, 121.8, 120.4, 119.1, 114.9,

83.7, 70.6, 55.5, 42.6, 39.4, 30.5, 29.1, 24.9, 23.9, 23.1, 17.2, 14.5, 14.1, 11.1. . λ_{max} (CH_2Cl_2)/nm: 285 (logε /dm^3 mol^{-1} cm^{-1}: 4.83), 297sh (4.77), 322 (4.75). MS (MALDI-TOF, DCTB) Anal.Calcd for $C_{59}H_{77}BrO_4$: 859.6 (25%), 860.6 (100%), 861.6 (64%), 862.6 (22%). Found: 859.6 (22%), 860.6 (100%), 861.6 (73%), 862.6 (21%). T_g = 51 °C.

Tris[(4-{7-[3,5-bis(4-{2-ethylhexyloxy}phenyl)phenyl]-9,9-bis-n-propyl-fluoren-2-yl})phenyl]amine **3**

A mixture of tri(4-iodophenyl)amine[6] (93.5 mg, 0.15 mmol), 7-[3,5-bis(4-{2-ethylhexyloxy}phenyl)phenyl]-2-(4,4,5,5,-tetramethyl-1,3,2-dioxaborolanyl)-9,9-bis-*n*-propylfluorene (646 mg, 0.75 mmol), tetrakis(triphenylphosphine)palladium(0) (26 mg, 0.02 mmol), toluene (15 mL), *t*-butanol (3 mL), and aqueous sodium carbonate (2 M, 7.5 mL) was placed under vacuum and then backfilled with nitrogen three times before being heated in an oil bath at 70 ºC for 48 h. After cooling to room temperature, the layers were separated, and the aqueous phase was extracted with ethylacetate (3 × 50 mL). The organic layers were combined, washed with brine (2 × 50 mL), dried over magnesium sulfate, filtered, and then the solvent was completely removed. The residue was purified in two steps: first, column chromatography over silica using a dichloromethane:*n*-hexane mixture (4:10) as eluent; second, the fractions containing the product were combined and further purified by chromatotron chromatography (2 mm silica plate) using dichloromethane:*n*-hexane mixtures (1.5:10 to 3:10) to afford **3** as a light yellow solid (210 mg, 57%). Elemental analysis (%) calcd for $C_{117}H_{207}NO_6$ C 87.0, H 8.5, N 0.6; Found: C 86.8, H 8.4, N 0.35. ^{1}H NMR (δ 500MHz, $CDCl_3$): 7.81 (3 H, brd, J = 4, FlH), 7.80 (3 H, brd, J = 4.5, FlH), 7.77 (6 H, d, J = 1.5, bpH), 7.71 (3 H, dd, J = 1.5, J = 1.5, bpH), 7.69 (3 H, dd, J = 1.5, J = 8, FlH), 7.65-7.68 (21 H, m, spH, FlH), 7.33 and 7.63 (12 H, brAA'BB', TPAH), 7.04 (12 H, 1/2AA'BB', spH), 3.90-3.96 (12 H, m, OCH_2), 2.03-2.10 (12 H, m, $PrCH_2$), 1.75-1.82 (6 H, m, EtCH), 1.32-1.60 (48 H, m, $EtCH_2$), 0.91-0.98 (36 H, m, $EtCH_3$), 0.75-0.86 (12 H, m, $PrCH_2$), 0.72 (18 H, t, J = 7, $PrCH_3$). ^{13}C NMR(δ 125 MHz, $CDCl_3$): 159.2, 151.8, 151.7, 146.7, 142.7, 142.1, 140.24, 140.20, 139.7, 139.5, 136.1, 133.6, 128.4, 127.9, 126.3, 125.6, 124.5, 124.4, 124.2, 121.7, 121.0, 120.1, 120.0, 114.9, 70.6, 55.5, 42.9, 39.4, 30.6, 29.1, 23.9, 23.1, 17.3, 14.6, 14.1, 11.1. λ_{max} (CH_2Cl_2)/nm: 284 (logε /dm^3 mol^{-1} cm^{-1}: 5.18), 323sh (4.92), 376 (5.15). MS (MALDI-TOF, DCTB) Anal.Calcd for $C_{117}H_{207}NO_6$: 2442.6 (52%), 2443.60(100%), 2444.6 (97%), 2445.6 (61%), 2446.6 (30%), 2447.6 (12%). Found: 2442.6 (41%), 2443.5 (100%), 2444.5 (91%), 2445.5 (63%), 2446.5 (31%), 2447.5 (11%). $T_{5\%}$ 417 °C. T_g = 107 °C (second scan at 100 °C/min). $E_{1/2(Ox)}$ = 0.3 V relative to the ferrocenium/ferrocene couple. PLQY (solution) = 69%.

Synthesis of 4

A mixture of tri(4-iodophenyl)amine (63 mg, 0.1 mmol), 7-[3,5-bis(4-{2-

ethylhexyloxy}phenyl)phenyl]-7'-(4,4,5,5,-tetramethyl-1,3,2-dioxaborolanyl)-9,9,9',9'-tetra-*n*-propyl-2,2'-bifluorene (550 mg, 0.50 mmol)[7], tetrakis(triphenylphosphine)palladium(0) (17 mg, 0.01 mmol), toluene (15 mL), *t*-butanol (3 mL), and aqueous sodium carbonate (2 M, 5 mL) was placed under vacuum and then backfilled with nitrogen three times before being heated in an oil bath at 70 ºC for 48 h. After cooling to room temperature, the layers were separated, and the aqueous phase was extracted with ethyl acetate (3 × 50 mL). The combined organic phases were washed with brine (2 × 50 mL), dried over magnesium sulfate, filtered, and then the solvent was removed. The residue was purified in two steps: first, column chromatography over silica using a dichloromethane:*n*-hexane mixture (6:10) as eluent; and finally, the fractions were separated by chromatotron chromatography (2 mm silica plate) dichloromethane:*n*-hexane mixtures (5:100 to 25:100) as eluent to give **4** as a light yellow solid (190 mg, 60%). Elemental analysis (%) calcd for $C_{234}H_{267}NO_6$ C 88.1, H 8.4, N 0.4; Found: C 87.95, H 8.7, N 0.5. ^1H NMR (δ 400 MHz, $CDCl_3$): 7.80-7.85 (12 H, m, FH), 7.77 (6 H, d, J = 1.5, bpH), 7.62-7.72 (45 H, m, spH, FH, bpH, TPAH), 7.34 (6 H, 1/2brAA'BB', TAPH), 7.04 (12 H, 1/2AA'BB', spH), 3.90-3.95 (12 H, m, OCH_2), 2.05-2.15 (24 H, brm, $PrCH_2$), 1.74-1.81 (6 H, m, EtCH), 1.31-1.61 (48 H, m, $EtCH_2$), 0.91-1.00 (36 H, m, $EtCH_3$), 0.79-0.90 (24 H, m, $PrCH_2$), 0.71-0.78 (36 H, m, $PrCH_3$). ^{13}C NMR(δ 100 MHz, $CDCl_3$): 159.2, 151.83, 151.78, 146.8, 142.7, 142.1, 140.6, 140.5, 140.3, 140.0, 139.97, 139.8, 139.5, 133.6, 128.4, 128.0, 126.2, 125.6, 124.5, 124.3, 121.8, 121.4, 121.1, 120.0, 119.9, 70.7, 55.6, 55.5, 42.9, 39.4, 30.6, 29.1, 23.9, 23.1, 17.4, 14.60, 14.58, 14.1, 11.1. λ_{max} (CH_2Cl_2)/nm: 263 (logε /dm^3 mol^{-1} cm^{-1}: 5.17), 280 (5.17), 353sh (5.28), 379 (5.38). MS (MALDI-TOF, DCTB) Anal.Calcd for $C_{234}H_{267}NO_6$: 3187.1 (30%), 3188.1 (78%), 3189.1 (100%), 3190.1 (86%), 3191.1 (53%), 3192.1 (28%), Found: 3187.2 (37%), 3188.2 (86%), 3189.2 (100%), 3190.2 (85%), 3191.2 (61%), 3192.2 (34%). $T_{5\%}$ 415 °C; T_g = 136 °C (second scan at 100 °C/min). $E_{1/2(Ox)}$ = 0.3 V relative to the ferrocenium/ferrocene couple. PLQY (solution) = 79%.

Absorbance and fluorescence measurements

For determining the molar extinction coefficients UV-visible spectra were recorded on a Varian Cary 5000 UV-Vis-NIR spectrophotometer in spectroscopic grade dichloromethane (CH_2Cl_2). For the fluorescence measurements all dendrimer solutions were prepared with spectroscopic grade tetrahydrofuran with a peak absorbance of less than 0.2. The absorbance spectra were recorded with a Varian Cary 5000 spectrophotometer. Fluorescence spectra for solutions were measured with a Horiba Jobin-Yvon Fluoromax 4 system with the dendrimer solutions excited at the peak of the absorbance.

Calculation of the analyte:dendrimer ratio

The analyte:dendrimer **3** ratio was calculated using a previously reported method that will be briefly outlined[8]. The difference in the areas under the SLD *versus* thickness curves for the pristine and saturated films is attributed to the additional scattering from the perdeuterated analyte that has penetrated into the film. As the SLD was modelled to be constant throughout the saturated films, the SLDs of the analytes were determined by assuming that their scattering density was distributed evenly throughout the film. The SLD of the swollen dendrimer matrix was then found by subtracting the analyte SLD from the total SLD of the saturated film. The dendrimer and analyte densities (in mol cm^{-3}) in the saturated films were calculated from the SLDs determined, the sum of the scattering lengths of the nuclei in the molecule, and Avogadro's number. Finally, the analyte:dendrimer ratios were then determined from the ratio of the molar densities.

Supplementary References

1. Yang, J. S. & Swager, T. M. Fluorescent porous polymer films as TNT chemosensors: Electronic and structural effects. *J. Am. Chem. Soc.* **120,** 11864–11873 (1998).
2. Cavaye, H. *et al.* Solid-State Dendrimer Sensors: Probing the Diffusion of an Explosive Analogue Using Neutron Reflectometry. *Langmuir* **25,** 12800–12805 (2009).
3. Wren, E. J. *et al.* The effect of dendronisation of arylamine centred chromophores on field effect transistor performance. *Polym. Chem.* **1,** 1117–1126 (2010).
4. Aldred, M. P. *et al.* Linearly polarised organic light-emitting diodes (OLEDs): synthesis and characterisation of a novel hole-transporting photoalignment copolymer. *J. Mater. Chem.* **15,** 3208–3213 (2005).
5. Lo, S.-C., Namdas, E. B., Burn, P. L. & Samuel, I. D. W. Synthesis and properties of highly efficient electroluminescent green phosphorescent iridium cored dendrimers. *Macromolecules* **36,** 9721–9730 (2003).
6. Mathias, L. J., Reichert, V. R. & Muir, A. V. G. Synthesis of rigid tetrahedral tetrafunctional molecules from 1,3,5,7-tetrakis(4-iodophenyl)adamantane. *Chem. Mater.* **5,** 4–5 (1993).
7. Cavaye, H. *et al.* Effect of Dimensionality in Dendrimeric and Polymeric Fluorescent Materials for Detecting Explosives. *Macromolecules* **43,** 10253–10261 (2010).
8. Shaw, P. E. *et al.* The binding and fluorescence quenching efficiency of nitroaromatic (explosive) vapors in fluorescent carbazole dendrimer thin films. *Phys. Chem. Chem. Phys.* **15,** 9845 (2013).
